# Higher radiation doses after partial laryngectomy may raise the incidence of pneumonia: A retrospective cohort study

**DOI:** 10.3389/fonc.2022.1072474

**Published:** 2022-12-27

**Authors:** Guoqi Lv, Xiuling Wu, Zhengying Wang, Kanglong Wu, Geer Ang, Shulin Cui, Yuqi Shi, Yu Wang, Delong Liu

**Affiliations:** ^1^ Department of Otorhinolaryngology Head and Neck Surgery, Dalian Municipal Central Hospital, Dalian, Liaoning, China; ^2^ Dalian Medical University, Dalian, Liaoning, China; ^3^ China Medical University, Shenyang, Liaoning, China; ^4^ The Second Hospital of Dalian Medical University, Dalian, Liaoning, China

**Keywords:** laryngeal cancer, radiation therapy, radiation dose, pneumonia, risk factors

## Abstract

**Background:**

Currently, studies have shown that a high dose of radiotherapy to the throat have various harmful and adverse effects on the patients’ laryngeal function, resulting in the development of pneumonia. This study aimed to explore how radiotherapy dose affected the probability of pneumonia following laryngeal cancer surgery.

**Materials and methods:**

A retrospective analysis was done on patients diagnosed with laryngeal cancer between 2010 and 2020 and were treated surgically and with postoperative radiotherapy in the same institution. This study included 108 patients in total, 51 of who were in the low-dose group and 57 of whom were in the high-dose group. Age, gender, the location of laryngeal cancer, the presence or absence of lymph node metastasis, and other demographic and clinical characteristics were collected, and the prevalence of postoperative pneumonia was compared between the two groups.

**Results:**

The total prevalence of postoperative pneumonia was 59.3%, but there was a significant difference between the two groups(high-dose group 71.9% VS low-dose group 45.1%; p=0.005). A total of 9.3% (10/108) of the patients had readmission due to severe pneumonia, and the rate of readmission due to pneumonia was significantly different between the two groups (high-dose group 15.8% VS low-dose group 2.0%, p=0.032). Additionally, the high-dose group’s prevalence of Dysphagia was significantly higher than the low-dose group’s. According to multivariate logistic modeling, high-dose radiation was a risk factor for pneumonia (OR=4.224, 95%CI =1.603-11.131, p=0.004).

**Conclusion:**

Pneumonia risk could increase with radiotherapy doses > 50 Gy in the treatment of laryngeal cancer. Therefore, we recommend that when the radiation dose surpasses 50Gy, doctors should pay particular attention to the lung health of patients with laryngeal cancer.

## 1 Introduction

One of the common malignancies of the head and neck is laryngeal cancer. Studies show that while its incidence has gradually increased in recent years. The survival rate has not increased (1). With the in-depth study of the disease, significant progress has been made in the treatment of laryngeal cancer. Although surgery has traditionally been the mainstay of laryngeal cancer treatment, other treatment modalities, such as radiotherapy, have also played an indispensable role (2). Especially for postoperative articulation function, local control, organ preservation, etc (3–5). However, radiotherapy for laryngeal cancer also inevitably has some toxic side effects, such as inflammatory reaction, Dysphagia, tissue fibrosis, etc (6). Additionally, because of the larynx’s complex anatomical structure and special location, the upper gastrointestinal tract and respiratory side effects caused by postoperative radiotherapy for laryngeal cancer are particularly obvious and seriously affect the quality of life of patients.

The radiation dose to the throat is crucial for the onset of hazardous and adverse effects from a dosimetric perspective. According to particular research, the severity of toxicity is significantly impacted by the increase in radiation dose (2). For instance, the radiation dose is positively connected with the larynx’s ability to swallow, the larynx’s muscle strength, and the frequency and intensity of mucositis (6–8). According to List et al. observed in one study, aspiration pneumonia may develop due to laryngeal swallowing function problems (9, 10). Additionally, numerous longitudinal studies have shown rates of inhaled pneumonia ranging from 2% to 65% within 3 months of radiation. At least 19% of non-cancer-related deaths are due to inhaled pneumonia (11).

There are no studies on the connection between postoperative radiotherapy for laryngeal cancer dose and pneumonia at this time. This study aimed to look into the connection between postoperative radiotherapy for laryngeal cancer dose and pneumonia incidence. We hypothesized that higher doses might increase the risk of pneumonia.

## 2 Materials and methods

### 2.1 Study sample

After approval by the ethics committee, patients with laryngeal cancer who underwent primary surgery and adjuvant radiotherapy in the Department of Otorhinolaryngology Surgery of our hospital from January 1, 2010, to October 1, 2020, were collected for a retrospective study. Inclusion criteria (1): Patients with laryngeal malignant tumors confirmed by pathology and treated with surgery; (2) The indication for radiotherapy was partial laryngectomy with incomplete resection margin. There were no contraindications to radiotherapy: ① Severe local tumor edema, necrosis and infection. ② Extensive invasion of the adjacent trachea, soft tissue or cartilage. ③ Large and fixed cervical lymph nodes with rupture. ④ patients with obvious laryngeal stridor, dyspnea and other respiratory obstruction symptoms. All patients received systemic treatment in the radiotherapy department of our hospital; (3) Age ≥18 years; (4) clear consciousness and stable condition; (5) Sign the informed consent; (6) No cognitive or communication disorders; (7) No serious chronic underlying diseases of heart, lung, liver or kidney or organ dysfunction. Exclusion criteria: (1) the patient refused to continue treatment if the treatment course was not completed; (2) patients who are intolerant to treatment; (3) patients who were lost to follow-up during treatment; (4) severe mental cognitive impairment; (5) hearing impairment; (6) severe cardiopulmonary dysfunction; (7) severe infection; (8) combined with organic diseases of the digestive tract affecting swallowing function; (9) complicated with neuromuscular diseases; (10) combined with other malignant tumors; (11) unable to cooperate with complete researchers. (12) patients with radiotherapy as a primary treatment; (13) patients not receiving radiotherapy; (14) patients with total laryngectomy.

### 2.2 Study design

Using the in-hospital BSWHIS electronic medical record system(Chuangyehuikang, Hangzhou, China), two otolaryngology doctors who were unaware of the study’s goal gathered the data for it. The imaging data (pulmonary CT) provided by the patients during the review was primarily used to confirm and record this study’s primary outcome measure (pneumonia). In addition, auxiliary diagnostic criteria include (1) body temperature; (2) purulent airway discharge; (3) Inflammatory test indicators. Imaging data before and after the two treatments were compared if the patient had a pneumonia diagnosis prior to radiation. We also regarded the outcome index as positive if there was gradual aggravation (The first pulmonary CT scan was taken before the radiotherapy; the second pulmonary CT scan was taken 6 weeks later, at the end of the radiotherapy course. We would like to clarify that the CT scan at the end of the radiotherapy course was a protocol that was developed during a previous project conducted in the radiotherapy department of our institution). What we need to state was that for those patients who showed the presence of new onset inflammatory lesions on pulmonary CT only but had no obvious clinical manifestation or a less severe clinical manifestation, we also considered them to develop the post radiotherapy pneumonia. Other demographic information and clinical traits of patients have also be gathered, such as radiation dose, age, gender, BMI, smoking and drinking history, history of illnesses (hypertension, diabetes), the location of laryngeal cancer (glottis, glottis, subglottic), type of surgery (Low-temperature plasma partial laryngectomy, open partial laryngectomy), whether it was accompanied by lymph node metastasis, whether or not concurrent chemotherapy. The concurrent chemotherapy protocol was as follows: cisplatin (100 mg/m^2^) or carboplatin (AUC=5), every 3 weeks (12). The overall clinical result of the trial was assessed using the swallowing performance score at the final follow-up (range, 0–4; 1 = normal swallowing; > 1 = Swallowing dysfunction) (6).

The radiation dose served as the primary factor in the grouping. This study was split into two groups based on radiation exposure: a high radiation dose group and a low radiation dose group (low radiation dose group ≤ 50Gy, included 64 patients; high radiation dose group>50Gy, included 67 patients). Previous studies have shown that aspiration pneumonia rarely occurs when the mean dose of laryngeal radiotherapy is less than 48Gy, and no cases of laryngeal stenosis occur when the mean dose is less than 53.9Gy (6). Therefore, 50Gy was defined as the cut-off value in this study. If the radiation dose was adjusted as part of a subsequent treatment regimen, the two data collection investigators recorded the dose at the higher dose. The setting of the patient’s irradiation field should be selected according to the different types of laryngeal cancer (glottic, supraglottic, and glottis descending). The range includes: Upper bound: hyoid level or lower edge of the hyoid bone or upper edge of the laryngeal notch or level of the first cervical vertebra; Lower bound: cricoid cartilage inferior edge level or near carina level; Anterior boundary: about 1-2 cm anterior to the anterior edge of the neck; Posterior border: anterior edge of the posterior laryngeal wall or anterior edge of cervical vertebrae or anterior and middle 1/3 junction of a cervical vertebral body or cervical transverse process; In addition, the lymph node area should be analyzed in combination with the location of lymphadenopathy and the need for prophylactic irradiation, such as superior glottic laryngeal cancer, which requires prophylactic irradiation of upper and middle cervical lymph nodes. In a word, the setting of the specific irradiation field should be adjusted appropriately according to the specific location of the tumor, the size of the lesion and the site of invasion.

### 2.3 Statistical analysis

The data analysis for this study used SPSS 25.0. The Kolmogorov-Smirnov test was performed to determine whether the measurement data were normal distribution. The mean and standard deviation were used to express a normal distribution; otherwise, the median (interquartile range) was used. The chi-square test evaluated the categorical variables, and the measurement data between the two groups were examined using the independent sample t-test or rank sum test. In order to create the regression model, many variables were incorporated into a logistic regression, and the OR value served as the benchmark for measuring the exposure factors. P <0.05 was regarded as statistically significant in this investigation.

## 3 Result

### 3.1 Demographic and clinical characteristics of the patients with laryngeal cancer

**Figure 1:**
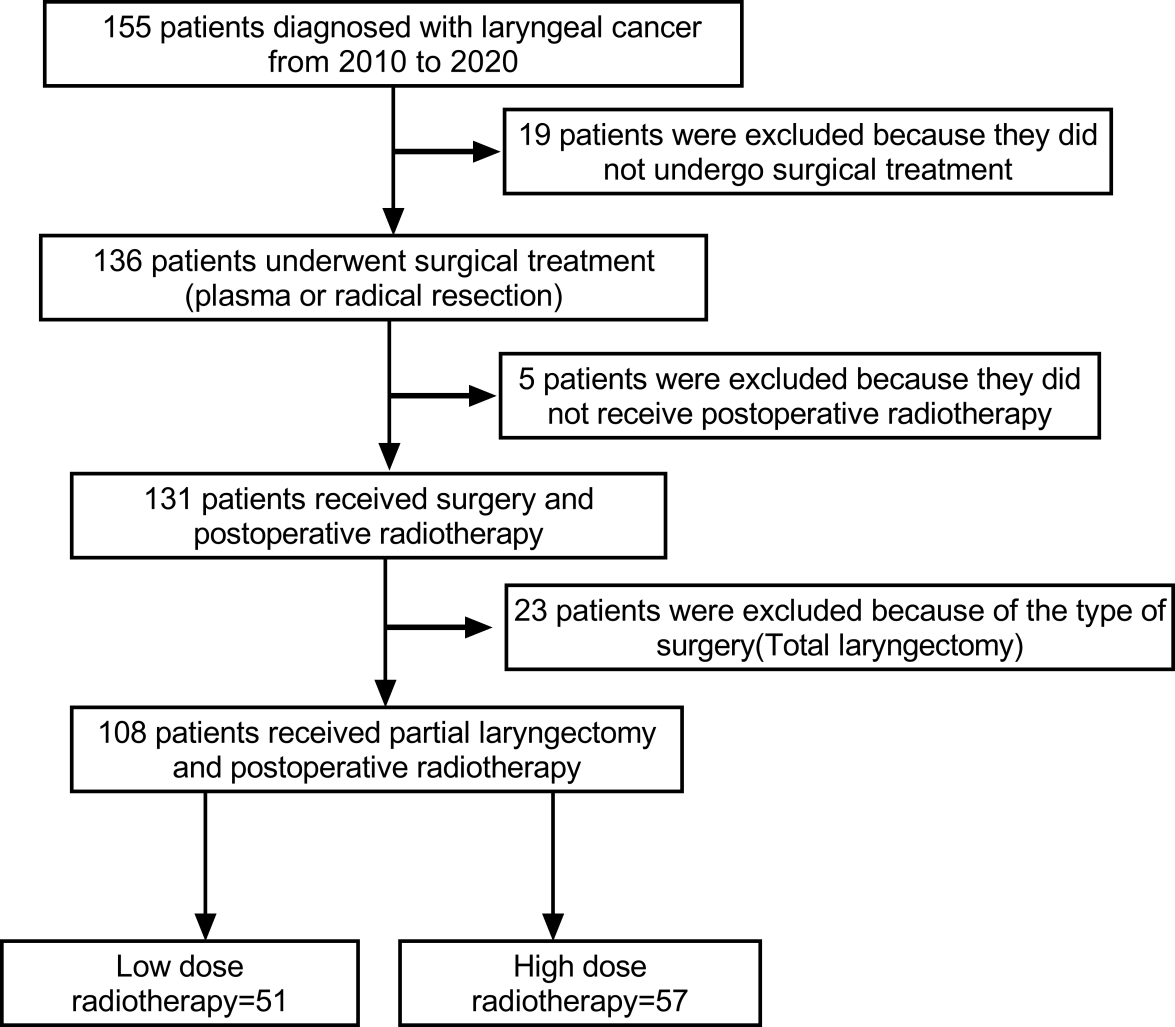
Flow chart of patients screening.

Patients, who were diagnosed with laryngeal cancer (glottic, supraglottic and subglottic) and received surgery and subsequent radiotherapy in the Department of Otolaryngology of our hospital from January 1, 2010, to October 1, 2020, were included. Initially, we included 155 patients. Based on inclusion and exclusion criteria, 19 patients were excluded because they did not undergo surgery, 23 patients were excluded because of total laryngectomy. In addition, 5 patients were excluded because they did not receive postoperative radiotherapy. Finally, 108 samples in total, 103 men (95.4%) and 5 women (4.6%), were included in this study (**Figure 1**). At admission, their average age was 63.61 ± 8.56. (range 44-85). With a mean follow-up of 6.3 ± 3.38 years, the study was followed up for at least two years (range 2.0-12.8).

### 3.2 Differences in the incidence of pneumonia and dysphagia between the high-dose and low-dose groups

**Figure 2:**
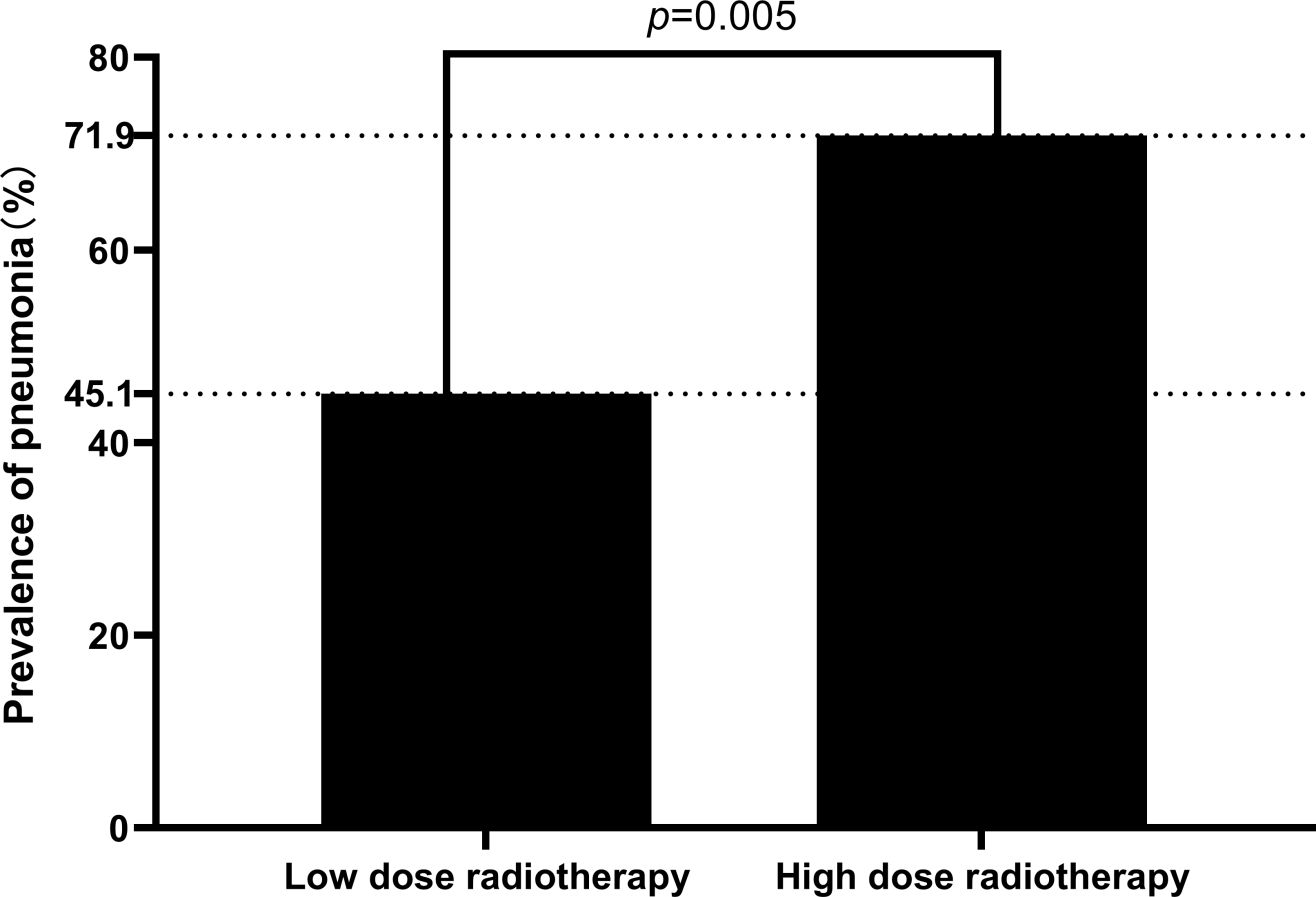
Differences in the prevalence of pneumonia between the two groups.

Age, gender, BMI, smoking, drinking history, history of diseases (hypertension, diabetes), did not statistically differ between the high radiation dose group and the low radiation dose group (p > 0.05). Between the two groups, the baseline was comparable (**Table 1**). In addition, there were no statistically significant differences in disease characteristics(location of laryngeal cancer, whether it was accompanied by lymph node metastasis, the size and extent of the tumor) and treatment characteristics(type of surgery, whether or not concurrent chemotherapy) between the two groups (**Table 2**). In this study, there were 10 patients receiving concurrent chemotherapy, 80.0% (8/10) and 20.0% (2/10) of whom received monodrug cisplatin and monodrug carboplatin respectively. In terms of surgical types, 61.1% (66/108) and 38.9% (42/108) patients underwent low-temperature plasma partial laryngectomy and open partial laryngectomy, respectively. In the high-dose group, the mean radiation dose was 64.39 ± 4.86Gy (range 54-70), with a median dose of 65.00Gy. The overall incidence of pneumonia following laryngeal cancer radiation was 59.3% (64/108). Surprisingly, however, there was a significant difference between the two groups in the prevalence of pneumonia, with only 22 patients in the low-dose group and 41 patients in the high-dose group. The prevalence of pneumonia in the high-dose group was substantially higher than that in the low-dose group(71.9% VS 45.1%, *p*=0.005) (**Table 3**; **Figure 2**). In addition, over the course of the follow-up period, Dysphagia (swallowing score > 1) was developed in a total of 55.6.0% (60/108) of the study’s patients. Notably, the likelihood of Dysphagia was significantly higher in the high-dose group than in the low-dose group (64.9% VS 45.1%, p=0.039) (**Table 3**). Additionally, pneumonia was present in up to 71.7% (43/60) of the individuals with Dysphagia.

**Table 1:** Demographic and clinical characteristics of different radiotherapy dose groups.

Category		Group		*p*-value
		Low dose radiotherapy	High dose radiotherapy	
Number	–	51	57	–
Age	–	63.27 ± 7.74	63.91 ± 9.29	0.701^*^
Sex	Male	48(94.1)	55(96.5)	0.899^▴^
	Female	3(5.9)	2(3.5)	
BMI	–	22.20 ± 2.61	22.40 ± 2.30	0.672^*^
Smoking	Yes	45(88.2)	49(86.0)	0.726^†^
	No	6(11.8)	8(14.0)	
Drinking	Yes	32(62.7)	32(56.1)	0.486^†^
	No	19(37.3)	25(43.9)	
Hypertension	Yes	12(23.5)	14(24.6)	0.900^†^
	No	39(76.5)	43(75.4)	
Diabetes	Yes	8(15.7)	5(8.8)	0.270^†^
	No	43(84.3)	52(91.2)	

Continuous data are specified as mean ± standard deviation; Categorical variables are defined as the number (percent).

BMI, body mass index.

^*^ T test; ^▴^Calibration chi square test; ^†^Chi square test.

**Table 2:** Disease characteristics and treatment characteristics of the two groups.

Category		Group		*p*-value
		Low dose radiotherapy	High dose radiotherapy	
Disease characteristics		-	-	-
The location of the laryngeal cancer	Glottic	43(84.3)	49(86.0)	0.885^#^
	Supraglottic	7(13.7)	8(14.0)	
	Subglottic	1(2.0)	0(0.0)	
Lymph node metastasis	Yes	12(23.5)	20(35.1)	0.189^†^
	No	39(76.5)	37(64.9)	
The size and extent of the tumor	T1	17(33.3)	13(22.8)	0.425^#^
	T2	20(39.2)	31(54.4)	
	T3	12(23.5)	12(21.1)	
	T4	2(3.9)	1(1.8)	
Treatment characteristics		-	-	-
Type of surgery	Low-temperature plasma partial laryngectomy	29(56.9)	37(64.9)	0.392^†^
	Open partial laryngectomy	22(43.1)	20(35.1)	
Concurrent chemotherapy	Yes	4(7.8)	6(10.5)	0.631^†^
	No	47(92.2)	51(89.5)	

Categorical variables are defined as the number (percent).

^#^Fisher test; ^†^Chi square test.

**Table 3:** Differences in the prevalence of pneumonia and swallowing scores between the two groups.

Category		Group		*p*-value
		Low dose radiotherapy	High dose radiotherapy	
Pneumonia	Yes	23(45.1)	41(71.9)	**0.005** ^†^
	No	28(54.9)	16(28.1)	
Swallowing Performance Scale	swallowing impairment(score >1)	23(45.1)	37(64.9)	**0.039** ^†^
	Normal swallowing(score ≤ 1)	28(54.9)	20(35.1)	

Categorical variables are defined as the number (percent).

^†^Chi square test.

Bold values denote statistical significance at the P < 0.05 level.

### 3.3 The outcome of pneumonia

Most of the patients diagnosed with post-radiotherapy pneumonia in this study had mild clinical symptoms and did not progress to severe pneumonia. However, 9.3% (10/108) of patients developed more severe pneumonia, leading to hospital readmission. There was a significant difference in the proportion of patients readmitted for pneumonia between the two groups (high-dose group=15.8% VS low-dose group=2.0%, *p*=0.032)(**Table 4**). 5 out of 10 patients who developed severe pneumonia eventually died from pneumonia (mortality = 50.0%).

**Table 4:** Clinical outcomes of pneumonia after radiotherapy.

Category	Group		*p*-value
	Low dose radiotherapy	High dose radiotherapy	
Not hospitalized	50(98.0)	48(84.2)	0.032^▴^
Readmission	1(2.0)	9(15.8)	

Categorical variables are defined as the number (percent).

^▴^Calibration chi square test.

### 3.4 The Results of multivariate regression

The multivariate logistic regression equation took into account variables such as radiotherapy dose, age, gender, BMI, history of smoking and drinking, history of previous illnesses (hypertension, diabetes), location of laryngeal cancer, type of surgery, whether it was accompanied by lymph node metastasis, whether or not concurrent chemotherapy was used, whether Dysphagia was present. According to the findings, receiving a lot of radiation therapy increased the incidence of pneumonia (OR=4.224, 95%CI=1.603-11.131, p=0.004) (**Table 5**).

**Table 5:** Results of binary logistic regression for pneumonia.

Variable		*p*-value	OR-value	95%CI
Age		0.866	0.994	0.930-1.063
Sex	Male	0.071	0.084	0.006-1.241
	Female^*^			
BMI		0.909	0.989	0.820-1.193
Smoking		0.873	0.886	0.202-3.882
Drinking		0.227	1.928	0.664-5.600
Hypertension		0.075	2.846	0.899-9.010
Diabetes		0.487	1.637	0.408-6.565
The location of the laryngeal cancer	Glottic	0.747	1.214	0.374-3.948
	Supraglottic			
	Subglottic			
Lymph node metastasis		0.161	0.403	0.113-1.434
The size and extent of the tumor	T1/T2/T3/T4	0.293	1.473	0.716-3.032
Type of surgery	Low-temperature plasma partial laryngectomy	0.107	2.561	0.815-8.048
	Open partial laryngectomy			
	Open total laryngectomy			
Concurrent chemotherapy		0.476	0.523	0.088-3.102
Radiotherapy dose	High dose radiotherapy	**0.004**	4.224	1.603-11.131
	Low dose radiotherapy^*^			
Swallowing Performance Scale	swallowing impairment(score >1)	**0.020**	3.716	1.225-11.270
	Normal swallowing(score ≤ 1)^*^			

*: Control group; OR, Odds Ratio; CI, confidence interval; BMI, body mass index.

Bold values denote statistical significance at the P < 0.05 level.

## 4 Discussion

The prevalence of pneumonia was significantly higher in patients receiving radiation doses > 50 Gy than in patients receiving doses ≤ 50 Gy, according to this study, and the risk of pneumonia in the high-dose group > 50 Gy was 4.224 times more than that in the low-dose group ≤ 50 Gy. Next, we have explored the reasons for this conclusion from the anatomical structure, physiological function, changes in the microenvironment, and metabolic disorders. First, radiation alters the throat’s anatomical structure. According to the larynx’s anatomical structure, which makes it a vital component of the respiratory tract, it performs a critical job safeguarding the lungs. Radiotherapy for laryngeal cancer can cause DNA double-strand breaks, resulting in reduced activity of essential proteins involved in DNA repair, resulting in direct damage to the throat mucosa and mucositis (13, 14). In a retrospective analysis, Brook et al. demonstrated a positive correlation between the frequency and severity of mucositis and the total radiation dosage (7, 8). The laryngeal mucosa undergoes repeated stimulation of inflammation, which results in hyperplasia and even scarring (15), narrowing the pharyngeal cavity.

Radiotherapy-induced laryngeal cartilage necrosis was initially documented in 1948 by Goodrich et al. (16). It has been shown by later research that radiotherapy may cause local tissue hypoxia, which can result in tissue fibrosis, endarteritis, vascular blockage, and decreased blood flow. The physiological function of the larynx is impacted, and the anatomical structure of the larynx is destroyed when microcracks in the perichondrium expose the deep surface cartilage to the air, resulting in infection and ultimately radionecrosis of cartilage (7, 17–19).

In summary, as an essential part of the respiratory tract, radiotherapy for laryngeal cancer can cause various changes in its tissue anatomy. Additionally, the degree of alteration becomes more severe as the radiation dose increases. We conclude that changes in laryngoparietal anatomy enhance the risk of pneumonia by increasing tissue exposure and airway resistance and that these changes and hazards increase with dose.

Second, radiation affects the throat’s normal physiological function. One of the essential defense mechanisms in the lung is the cough reflex. To ensure that the tumor bed and regional lymph nodes receive sufficient radiation doses, radiotherapy typically encompasses the throat, trachea, and a broader area of the head and neck. However, radiotherapy may cause laryngeal muscle atrophy and weakness, as well as decreased levels and changes in the distribution of the neuropeptide substance P (SP) and vasoactive intestinal peptide (VIP) (20, 21). These effects may lead to decreased throat motor strength (22). We believe that these factors combine to suppress the cough reflex. A 2007 retrospective study showed that the cough reflex is frequently inadequate in patients who received radiotherapy for head and neck cancer, failing to cough up phlegm built up in the throat and adequately protect the respiratory system following radiation (23).

For the physiological function of the throat, dysfunctional swallowing is a significant issue that patients are concerned. Radiotherapy dose and pharyngeal swallowing function were positively associated in retrospective research by Caglar et al. in 2008 (6). In this study, 71.7% (43/60) of the 108 patients had pneumonia, while 55.6% (60/108) of the patients had Dysphagia. These findings imply that Dysphagia may contribute to the emergence of pneumonia following postoperative radiotherapy for laryngeal cancer. Here’s how we clarify: First, an increase in radiation dose can cause excessive activation of TGF-1 (24) (a peptide involved in collagen deposition and degradation), and the imbalance of collagen deposition degradation leads to fibrosis of throat tissue, which causes abnormal swallowing muscle movement and decreases swallowing function in patients (25). Second, patients’ laryngeal gland distribution is altered due to neck radiotherapy, which lowers the serous/mucous adenocyte ratio (26). This weakens the local defense function of the larynxlarynx’s ability to defend itself locally and makes saliva output stickier. The patient also has an atypical cough reflex, which adds to the difficulty swallowing. Patients with Dysphagia often have food residue that cannot enter the esophagus smoothly and cause aspiration. Compared with patients without Dysphagia, patients with Dysphagia have a greater residual rate and a higher inhalation rate. A prospective study by Feng et al. found that whether the radiation dose of the pharyngeal contractile muscles and some larynx structures was greater than 50Gy was significantly associated with aspiration (27). This finding is similar to our study, in which 71.9% of the patients in the high-dose group developed pneumonitis after radiotherapy.

We, therefore, proposed that one of the significant causes of pneumonia following postoperative radiotherapy for laryngeal cancer was decreased cough reflex, and Dysphagia brought on by decreased laryngeal muscle strength, tissue fibrosis, and aberrant distribution of local glands.

Third, the microbiota of the upper respiratory tract is harmed by changes in the microenvironment. The term “microbiota” describes the assortment of microorganisms in a specific environment (28). The microbiota of the upper respiratory tract and the lung are similar in humans (29, 30). According to some researchers, the oropharynx is the primary source of lung bacteria. Vesty et al. suggested that when the radiation dose was 40-60Gy, the oral microbiota was dysregulated, significantly increasing the incidence of oral mucositis (31). Both direct mucosal dispersion and microaspiration from the upper respiratory tract are routes for bacteria in the oropharynx to enter the lungs (32, 33). Recent research has revealed that the microbiome’s diversity declines as radiation exposure increases (28, 34, 35).

Inflammation from radiation-induced alteration of the microbiota is hypothesized to be brought on by two main mechanisms (36, 37). First, radiation exposure causes tissue oxidation and inflammation directly, which alters the local microenvironment and results in ecological dysregulation. Second, radiation can harm cellular DNA and RNA, causing apoptosis, which results in ulceration and causes toxic damage to the epithelium. The results in the exposure of tissues that should not come into contact with bacteria, resulting in an inflammatory response that is translocated and colonized, further increasing these responses (38).

Based on the concepts mentioned above, it is conceivable that the increased radiation dose of postoperative laryngeal cancer can further decrease the diversity of respiratory microbiota, causing the imbalance between beneficial and harmful microbiota to worsen and raising the risk of pneumonia.

Fourth, metabolic diseases are brought on by radiotoxicity. Radiotoxicity has been researched more thoroughly as molecular radiopathology and clinical radiobiology have advanced. Radiation-induced fibrosis, atrophy, vascular damage, and endocrine disorders are all examples of radiotoxicity. According to current thinking, radiotoxic damage has two mechanisms: direct loss of parenchymal cells, and the loss of vascular endothelial cells (39). Tissue destruction is a dynamic and progressive process. Evidence supports the idea that tiny dose adjustments can result in significant changes in toxicity (40). The deposition of radiotoxicity induces DNA damage and changes in the cellular microenvironment through chemokines, inflammatory cytokines, fibrotic cytokines, altered cell-cell interactions, the influx of inflammatory cells, and repair processes (39). However, DNA damage and changes in its repair function play an essential role in developing normal tissue damage. In a prospective study by Popanda et al. in 2003, DNA repair capacity was significantly reduced at total radiation doses > 50.4 Gy, in which 81 of 87 breast cancer patients developed acute skin damage (41). So, it is reasonable to believe that the radiation toxicity of postoperative radiotherapy for laryngeal cancer could increase significantly when the dose is more than 50Gy.

For the range of radiation toxicity, the early classical theory is the target cell hypothesis: The leading cause of the toxic side effects of radiotherapy is functional defects caused by the direct killing of the target cells (42). However, the research by Brock, W. A. et al. revealed that the “bystander effect”—when unirradiated cells around the target cell are killed—can occur when a single cell is exposed to a high-precision microbeam of radiation (43). Additionally, the toxicity becomes more severe when the radiation dose rises.

We consider that the throat and lungs are a part of the respiratory system and that the larynx’s radiation toxicity during radiotherapy for laryngeal cancer could invariably harm the nearby airway, resulting in the development of pneumonia. Additionally, if the dose is increased, the toxicity could change more significantly, raising the risk of pneumonia.

Studies have shown that high radiation doses increase the incidence of pneumonia, and the radiation dose of patients who die of aspiration pneumonia is significantly higher than that of patients who die of other causes (11). According to the results of this study, 90.7% of patients with pneumonia after radiotherapy had mild clinical manifestations. But in our view, we cannot ignore pneumonia as a complication of radiotherapy. Because 9.3% (10/108) of patients in this study were readmitted to hospital due to severe pneumonia, and up to 50.0% (5/10) of them died due to severe pneumonia. This result deserves our attention. Therefore, we believe that timely interventions are warranted for those patients at increased risk of aspiration, such as postural techniques, improved sensory input, swallowing rehabilitation, and dietary changes that can reduce the incidence of pneumonia and associated mortality (44). At present, some researchers are exploring ways to reduce the side effects of radiotherapy, such as radioprotective agents (amifostine and palifermin, etc), and emphasizing that radiotherapy provides a highly suitable dose distribution around the tumor target and avoids mucosal and salivary gland damage as much as possible (45). However, there is substantial heterogeneity among current studies on radioprotective agents, which is unable to demonstrate the benefit of radioprotective agents for patients (46). So we think that more studies on radioprotective agents are needed in the future to prove their effectiveness.

The surgical treatment of laryngeal cancer requires not only the removal of the tumor itself but also the removal of part of the normal tissue to retain enough safe margins to reduce recurrence. Postoperative radiotherapy can cause scar formation or atrophy, affecting the larynx’s normal anatomical structure and physiological function. Although the results of this study show that the surgical approach does not increase the incidence of postoperative pneumonia, the possibility of an increased risk of pneumonia due to surgical changes in the normal anatomy of the larynx, muscle movement, and abnormal mucosal secretion cannot be excluded. More studies are needed to prove the relationship between surgery and pneumonia in laryngeal cancer patients in the future. The study includes some limitations. First, since this study is retrospective, there may be limitations and inaccurate data. Second, the study’s sample size is limited, which could result in some random errors. To discover the ideal dose of radiotherapy to prevent the complication of pneumonia while taking into account the therapeutic effect, we would like to underline the necessity for prospective research with bigger sample sizes and a more granular grouping of radiation doses

## 5 Conclusion

In this study, we found that the risk of pneumonia during postoperative laryngeal cancer treatment increased with radiation doses of more than 50 Gy. The development of pneumonia impacts patients’ quality of life and the long-term mortality of pneumonia brought on by radiotherapy. In order to improve patients’ quality of life, we recommend that doctors pay special attention to their patients’ lung health and promptly evaluate and actively intervene when the radiation dose exceeds 50 Gy.

## Data availability statement

The raw data supporting the conclusions of this article will be made available by the authors, without undue reservation.

## Ethics statement

The studies involving human participants were reviewed and approved by ethics committee of Dalian Municipal Central Hospital, Liaoning Province, China. The patients/participants provided their written informed consent to participate in this study.

## Author contributions

All authors contributed to the study conception and design. Material preparation, data collection and analysis were performed by GL, ZW, XW, GA, KW, SC, YS and YW. The first draft of the manuscript was written by GL and all authors commented on previous versions of the manuscript. All authors contributed to the article and approved the submitted version.
